# Early detection of an epidemic erythromelalgia outbreak using Baidu search data

**DOI:** 10.1038/srep12649

**Published:** 2015-07-28

**Authors:** Yuzhou Gu, Fengling Chen, Tao Liu, Xiaojuan Lv, Zhaoming Shao, Hualiang Lin, Chaobin Liang, Weilin Zeng, Jianpeng Xiao, Yonghui Zhang, Cunrui Huang, Shannon Rutherford, Wenjun Ma

**Affiliations:** 1 Guangdong Provincial Institute of Public Health, Guangdong Provincial Center for Disease Control and Prevention, Guangzhou, China; 2 Chancheng Prefectural Center for Disease Control and Prevention of Guangdong Province, Foshan, China; 3 Guangdong Provincial Center for Disease Control and Prevention, Guangzhou, China; 4 Public Health School of Sun Yat-sen University, Guangzhou, China; 5 Center for Environment and Population Health, School of Environment, Griffith University, Brisbane, Australia

## Abstract

Dozens of epidemic erythromelalgia (EM) outbreaks have been reported in China since the mid-twentieth century, and the most recent happened in Foshan City, Guangdong Province early 2014. This study compared the daily case counts of this recent epidemic EM outbreak from February 11 to March 3 with Baidu search data for the same period. After keyword selection, filtering and composition, the most correlated lag of the EM Search Index was used for comparison and linear regression model development. This study also explored the spatial distribution of epidemic EM in China during this period based on EM Search Index. The EM Search Index at lag 2 was most significantly associated with daily case counts in Foshan (*ρ* *=* *0.863, P* *<* *0.001*). It captured an upward trend in the outbreak about one week ahead of official report and the linear regression analysis indicated that every 1.071 increase in the EM Search Index reflected a rise of 1 EM cases 2 days earlier. The spatial analysis found that the number of EM Search Indexes increased in the middle of Guangdong Province and South China during the outbreak period. The EM Search Index may be a good early indicator of an epidemic EM outbreak.

Erythromelalgia (EM) is a clinical syndrome characterized by a triad of erythema, burning pain and increased temperature of feet or hands or both. This syndrome is rare in the western world^1^,^2^, and there are no outbreak reports in developed countries. More than 70 epidemic EM outbreaks and over 80,000 cases have been reported in Chinese literature since the mid-twentieth century. For example, around 10,000 and 19,000 cases were observed in Hunan and Hubei Province respectively during a serious outbreak in 1987, and the number of cases in Fujian Province and Hainan Province were more than 10,000 and 11,000 in 1990 outbreak, respectively^3^,^4^,^5^,^6^,^7^,^8^,^9^. Although the onset of epidemic EM is acute, its clinical symptoms are not very serious and usually disappear within a few days^7^,^8^,^10^. As China has not developed any traditional disease surveillance to monitor this syndrome, the real situation of epidemic EM outbreaks remains unknown in China.

Most epidemic EM outbreaks in China have been reported between February and March, coinciding with a V-shaped temperature change: namely a sharp temperature decline followed by a rapid temperature rise within a few days. Previous studies have hypothesized that these large temperature fluctuations that occur in South China are associated with epidemic EM outbreaks^6^,^7^,^8^,^11^,^12^,^13^,^14^. Furthermore, Liu *et al.*^14^ recently found that one degree Celsius increment of daily temperature might trigger an average rise of 1.22 EM cases in epidemic EM outbreak. During February 2014, the temperature in Foshan City of Guangdong Province experienced a very large temperature fluctuation, accompanied by an epidemic EM outbreak in two high schools. The cases of this outbreak were characterized by burning pain and numbness in toes and feet. As most cases were mild, and epidemic EM is not a notifiable disease in China, it is not clear whether unreported cases occurred elsewhere in Guangdong or China during this period.

The availability and popularity of the Internet has grown greatly in recent years. As at December, 2013, there were 618 million Internet users in China, accounting for about 45.8% of the national population, and the proportion in Guangdong Province was even higher^15^. At the same time, an increasing number of people, including patients and their family members, are inclined to search online for health information before seeking medical service^16^,^17^, making it possible to monitor the health status of the population by tracking changes in frequencies of specific search keywords. Internet search engines are now the most common tool to obtain information for Internet users^18^,^19^, and data from different search engines have been successfully utilized for early detection of diseases such as influenza and dengue^17^,^18^,^20^,^21^,^22^,^23^,^24^,^25^,^26^. Such studies suggest that Internet search data-base surveillance might be a novel way to monitor epidemic EM outbreaks in near real-time.

Baidu is the most popular search engine in China, with 86.7% of Internet users preferring it^19^. This wide use makes it the most representative for analyzing Chinese online behavior^26^. Further, the search volume of Baidu users are released daily on Baidu’s Index website (http://index.baidu.com), which allows for timely capture in the changes in search keywords. Although more and more studies are investigating the relationship between search data and some infectious diseases, no study has yet focused on epidemic EM. Due to the lack of a traditional surveillance system, the Internet surveillance approach for early detection of epidemic EM outbreaks is a promising one.

The present study compared Baidu search data with case counts reported in the Foshan outbreak during the same period, in order to identify whether there was an association between epidemic EM and Internet search behavior, and develop an Internet search data-based surveillance method which would be useful to detect an outbreak of epidemic EM in the future.

## Materials and Methods

### Data sources

#### Outbreak data

This study used daily case counts over the entire 21 day outbreak period from February 11 to March 3, 2014 in Foshan City of Guangdong Province, China. The definition of a case is that a student in an outbreak high school reported an onset of pain, redness or numbness in toes or feet with no obvious cause after February 10, 2014. We eliminated those cases induced by injury. The first case was reported on February 27 and 494 cases were retrospectively confirmed by epidemiologists and clinical experts after a systematic field investigation. Daily case counts are shown in Table 1.

#### Baidu search data

The Baidu index website (http://index.baidu.com) contains search volumes for numerous keywords keyed in by Baidu users from June 2006. Data are available on a daily basis, at a city, province and national level. Considering the time lags between symptoms onset and online searching, we collected the data for 24 days from February 11 to March 6, 2014. The search volume for the same period in 2013 was collected for comparison.

#### Meteorological data

Due to the hypothesis of an association between epidemic EM and large temperature fluctuation we collected the daily maximum temperature in Foshan City from February 6 to March 3, 2014 (a total of 26 days), which contains the entire period of a large temperature change. Meteorological data were obtained from a free weather query website in Chinese (http://www.tianqihoubao.com).

No ethics committee approval or written consent from patients were required to obtain since only daily count data was obtained, and no information about the identity of any case was revealed.

### Keyword selection and filtering

Keyword selection is the critical issue in Internet search data-based surveillance, as it directly affects the ability and detective accuracy of the surveillance method. Different people may type in entirely different words when searching the same information, especially when searching in Chinese language, where one meaning can be expressed in several ways. Consequently, diverse results can be obtained by selecting different keywords. Despite the significance of this, there are no principles or standards for guidance^18^,^26^,^27^. Previous studies generally chose the names or clinical symptoms of target diseases as their core keywords^22^,^23^,^25^,^26^. As EM is a little-known disease within the lay Chinese community, insufficient search volume of this word leads to Baidu’s failure in calculating its search information. Therefore, we chose primary keywords which represent the major clinical characteristics of cases in 2014 and previous outbreaks (see Supplementary Table S1). A Chinese website (http://tool.chinaz.com/baidu/words.aspx) was used for further obtaining related keywords. Related key-word recommendations in the website not only include suggestions from Baidu, but also mining from portal websites, blogs, and online reports using semantic correlation analysis^26^. Upon typing in the 19 primary terms respectively, we obtained 62 related keywords (see Supplementary Table S1).

However, more keywords do not necessarily lead to a better result^17^,^28^ since some recommended keywords are not closely related to EM, which could reduce the detective ability of the surveillance system. Hence, we collected the search data in Foshan City from Baidu and filtered keywords following two steps:

1. We eliminated the words irrelevant to EM and those with a search volume of zero during the outbreak period, and 32 keywords remained (see Supplementary Table S1).
2. Spearman’s rank correlation coefficients (ρ) were then calculated between daily case counts and daily search volumes for each keyword using different time lags. We deleted the words with maximum correlation coefficients less than 0.4 in each time lag and those correlations that were statistically insignificant. Taking into account the remaining number, as well as strength of the correlation of keywords that met the criteria above, we considered time lags of 0 to 3 days. The remaining keywords for each of the four time lags were 14, 15, 17 and 17, respectively (Table 2).

### EM Search Index composition

Following selection and filtering, the remaining keywords were used for composition of an EM Search Index for each time lag. Weights of keywords were defined by the strength of the correlation coefficient^26^,^27^. The weights calculation and EM Search Index composition formulae are as follows:
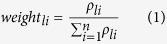

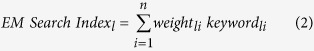
In the above formulae, *l* denotes time lag of the search data, *n* is the number of keywords at each time lag, *keyword*_*li*_ and *weight*_*li*_ represent the i^th^ keyword and the weight of it.

### Epidemic EM outbreak detection

In order to compare the epidemic situation with temperature change, we first graphed a line figure to depict the relationship between daily maximum temperature and daily case counts.

Spearman’s rank correlation coefficients were then calculated between outbreak data and the EM Search Index for each time lag of 0 to 3 days. The time lag that has the largest coefficient was selected for further analysis. Based on this, we further developed a linear regression model as follows:
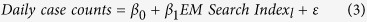
*EM Search Index*_*l*_ denotes the lag EM Search Index with the largest correlation, *β*_1_ as the regression coefficient. The model estimates the case count *l* days before, based on the Baidu search data for the current day.

Though the temperature fluctuation between February and March 2014 was widespread in South China, there were no other reports of epidemic EM outbreaks from other cities or provinces. Therefore, it was not clear whether EM cases occurred elsewhere during this period. We calculated the EM Search Index from February 11 to March 3, 2014 for Guangdong Province and 33 other provinces/municipalities of China. By plotting these data on maps, we aimed to roughly explore whether similar outbreaks of epidemic EM occurred in other parts of China during this period. In order to understand the influence of regional difference, Internet search data from February 11 to March 3 2013 were collected for comparison.

All analyses were performed using SPSS 19.0, and the maps were plotted with ArcGIS 9.3 (ESRI).

## Results

**Figure 1:**
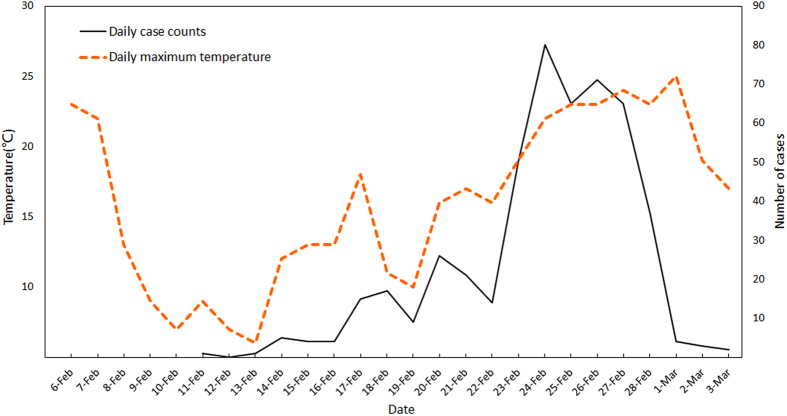
Daily case counts and daily maximum temperature. This figure displays the pattern of temperature change in Foshan City between February and March 2014 and provides the trend in daily EM case counts within this period.

A large fluctuation in temperature in Foshan City between February and March in 2014 was observed. Daily maximum temperature suddenly dropped about 10 °C on February 8 and continuously declined to the lowest (6 °C) on February 13, then slowly returned to a relatively high level afterwards (Fig. 1). When temperature began to increase, EM cases occurred (Fig. 1). Spearman’s rank correlation coefficient analysis showed that the daily case counts were positively associated with daily maximum temperature during the temperature increase (*ρ* *=* *0.650, P* *=* *0.001*).

EM Search Indexes for time lags of 0 to 3 days were composed of 14, 15, 17 and 17 keywords respectively (Table 2), and the correlation coefficients between EM Search Indexes and outbreak data are listed in Table 3. We found the correlation getting closer with the increase of lag days before reaching a peak at lag 2 (*ρ* *=* *0.863, P* < *0.001*). Therefore, EM Search Index at lag 2 was chosen for further analysis.

**Figure 2:**
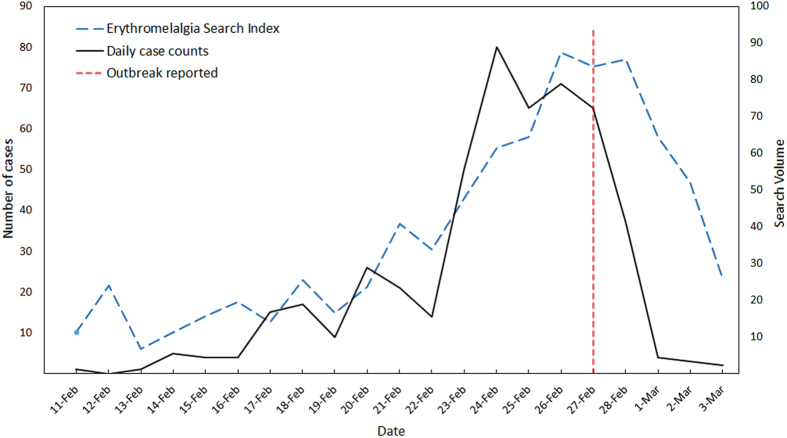
EM Search Index and daily case counts. This figure describes the changes in daily EM case counts and the EM Search Index at lag 2 during the outbreak period (February 11–March 3) for Foshan City. The report date of outbreak is clearly indicated.

We then graphed the curves of daily case counts and EM Search Index at lag 2 over the outbreak period (Fig. 2). Obviously, the search data accurately captured the change in daily case counts. Particularly, we found an apparent increase in EM cases after February 20, followed by a similar uptrend of search volume after February 21. However, the first case hadn’t been reported until February 27 by the local Center for Disease Control and Prevention, which suggested that, although there was a lag between the EM outbreak and the EM Search Index, the EM Search Index still had the ability to detect the epidemic about 1 week before the outbreak was reported.

The coefficient (*β*_1_) for the linear regression model between outbreak data and the EM Search Index was 0.934 (*P* < *0.001*), indicating that during the outbreak period, every 1.071 increase in EM Search Index reflected a rise of 1 case 2 days before. The R^2^ was 0.83, suggesting that the Search Index could explain 83% of the variation in daily case counts.

**Figure 3:**
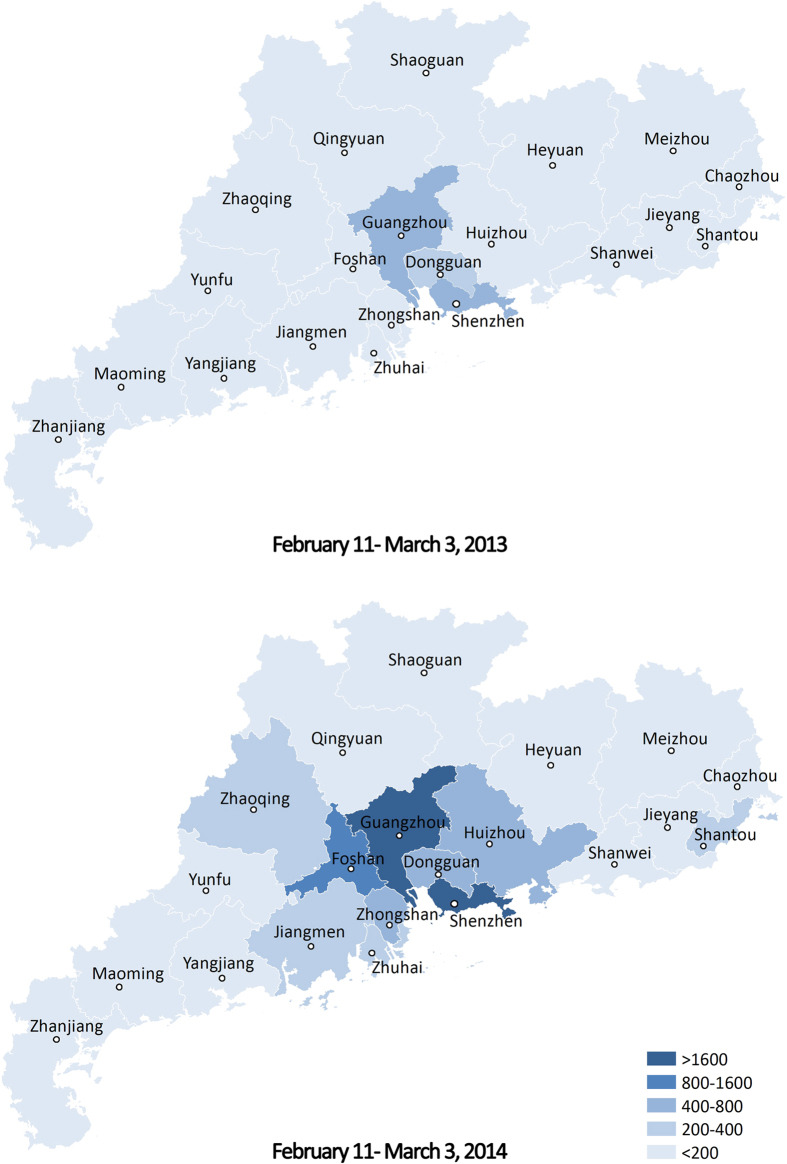
The spatial distribution of EM Search Index in Guangdong Province, China. This figure depicts the spatial distribution of EM Search Index counts across Guangdong Province during the outbreak period in Foshan City in 2014 by filling different colour depth for the cities through ArcGIS 9.3 (ESRI). Distribution of the same period in 2013 was plotted for comparison.

**Figure 4:**
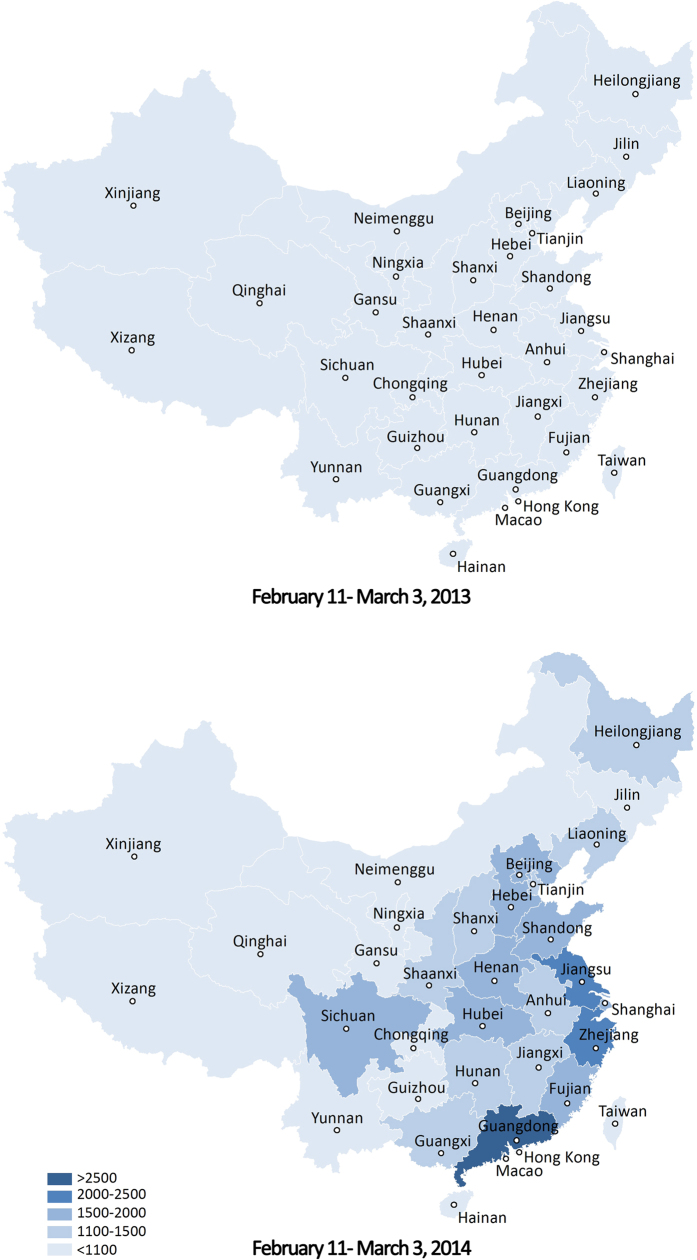
The spatial distribution of EM Search Index in China. This figure depicts the spatial distribution of EM Search Index counts across China during the outbreak period in Foshan City in 2014 by filling different colour depth for the provinces/municipalities through ArcGIS 9.3 (ESRI). Distribution of the same period in 2013 was plotted for comparison.

The EM Search Index from February 11 to March 3, 2014 for each city in Guangdong Province and 34 provinces/municipalities in China were plotted on maps, in contrast with the same period of 2013 (Figs 3 and 4). As demonstrated in Fig. 3, most cities of Guangdong Province showed low search frequencies in 2013, but a much higher EM Search Index was observed in Guangzhou, Foshan and Shenzhen in 2014. South China and East China showed relatively high EM Search Index in 2014, with the highest in Guangdong. In contrast, no region showed a high EM Search Index during the same period in 2013 (Fig. 4).

## Discussion

Since Eysenbach *et al.*^20^ set the important precedent for disease surveillance using Internet search data, there have been more and more studies on this topic. Most existing studies have focused on infectious diseases such as influenza and dengue fever^17^,^21^,^22^,^23^,^24^,^25^,^26^. This study is the first that has investigated the application of Internet search data in the early detection of outbreaks of epidemic EM.

In this study, we compared Baidu search index counts and daily case counts of a recent epidemic EM outbreak in Foshan City, Guangdong Province, China, and found that the EM Search Index at 2 lag days was significantly associated with an EM outbreak, and every 1.071 increase in EM Search Index might reflect a rise of 1 case 2 days before. These findings indicate that the onset of EM symptoms were associated with an increase in Internet search behavior for keywords relating to the illness after 2 days. Even though a 2 days lag was identified, the EM Search Index captured the sharp uptrend of daily case counts about a week ahead of the official report because of the delayed reports from the local Center for Disease Control and Prevention, This suggests that EM Search Index may be a good predictor for early detection of epidemic EM outbreaks. Due to little attention to EM by the public, no mass media reported EM during this outbreak, which adds weight to the utility of Internet search data and how it reflects individual’s health concerns and issues^29^. However, epidemic EM have mainly occurred in students living in schools, which makes our results useful for extrapolating to a similar population rather than general population^14^.

EM is little known within the Chinese ordinary people and insufficient knowledge might result in more pain and more panic during an epidemic EM outbreak. An early detection system could help to facilitate the timely treatment of cases and ease public concerns about the health symptoms. Previous studies reported the phenomenon of epidemic EM outbreaks accompanied by a large temperature fluctuation^6^,^7^,^8^,^11^,^12^,^13^,^14^, and this is confirmed by the outbreak that happened in Foshan in February and March, 2014. Certainly, conducting EM surveillance in schools, communities or hospitals during large temperature fluctuations is a direct way to detect EM outbreaks. However, it may be more cost-effective to monitor the changes of temperature and EM Search Index simultaneously. For example, coinciding with the sharp drop in ambient temperature followed by a temperature rise within a short period, we observed an obviously increasing trend of EM Search Index, which could be a strong signal for the occurrence of an EM outbreak. Therefore, Internet search data provides an opportunity for government or the public to early detect epidemic EM outbreaks and consequently take measures in time.

According to the Chinese literature, most epidemic EM outbreaks have coincided with large temperature changes in many provinces of South China^3^,^4^,^5^,^6^,^8^,^9^,^30^,^31^,^32^,^33^,^34^,^35^. For example, the outbreak in 1987, which affected Hubei, Henan, Hunan, Jiangxi and Zhejiang Province of South China^4^,^5^,^6^,^7^,^31^,^32^, and up to six provinces including Fujian, Anhui, Guangdong, Guangxi, Guizhou and Hainan of South China were involved in the outbreak^3^,^4^,^8^,^9^,^30^,^33^,^34^,^35^,^36^. Thus it is possible that the EM outbreak might not only limited in Foshan City in 2014 because ambient temperature fluctuation could be observed in many parts of South China between February and March. Therefore, we tried to retrospectively explore spatial distribution of epidemic EM using EM Search Index. Our results showed that cities in the middle of Guangdong Province and some provinces in South China had relative high search frequencies on symptoms of EM during the outbreak period. Some of the cities or provinces with high EM Search Index have ever occurred one or even more than one epidemic EM outbreaks in previous studies, such as Guangzhou^13^,^36^, Shenzhen^37^, Zhongshan^38^ of Guangdong Province and Zhejiang^31^, Jiangsu^39^, Henan^4^, Fujian^3^,^9^, Hebei^40^, Hubei^4^,^6^ and Hunan^4^,^5^,^12^. On the other hand, the whole country showed a low search frequency during the same period in 2013, when there was no large temperature fluctuation, suggesting that there was a real epidemic during this time. From our findings, we speculate that these cities or provinces with greater EM Search Index counts might have experienced epidemic EM outbreaks during this period in 2014, when the temperature experienced a large fluctuation.

There are some limitations of this current study. First of all, Baidu doesn’t release the search data of keywords without sufficient search volume, which might result in an underestimation of correlation. Additionally, although the selected keywords captured the trend of outbreak data very well, there still may be some omission due to the diversity of online search habits, and we haven’t got other data for model validation. Thirdly, a number of factors affect the individual search behavior thereby influencing the sustainability of our detection model^20^,^23^,^25^. Also, Internet access is uneven throughout China, with the lowest provincial Internet penetration of 32.6% in Jiangxi Province and the highest (75.2%) in Beijing (Internet penetration of each province/municipality of mainland China in 2013 see Supplementary Table S2), and the population sizes of different regions are also different. Thus, the accuracy of comparison of actual search index counts between cities or provinces should be considered with caution.

In conclusion, the EM Search Index using Baidu search term methodology may be a good indicator for early detection of an epidemic EM outbreak, especially when combined with temperature change monitoring.

## Additional Information

**How to cite this article**: Gu, Y. *et al.* Early detection of an epidemic erythromelalgia outbreak using Baidu search data. *Sci. Rep.* **5**, 12649; doi: 10.1038/srep12649 (2015).

## Supplementary Material

Supplementary Information

## Figures and Tables

**Table 1:** Daily EM case counts during the outbreak period in Foshan City.

**Date**	**Case count**	**Date**	**Case count**	**Date**	**Case count**
2014-02-11	1	2014-02-18	17	2014-02-25	65
2014-02-12	0	2014-02-19	9	2014-02-26	71
2014-02-13	1	2014-02-20	26	2014-02-27	65
2014-02-14	5	2014-02-21	21	2014-02-28	37
2014-02-15	4	2014-02-22	14	2014-03-01	4
2014-02-16	4	2014-02-23	50	2014-03-02	3
2014-02-17	15	2014-02-24	80	2014-03-03	2

**Table 2:** Keywords under time lags of 0 to 3 days after second step filtering.

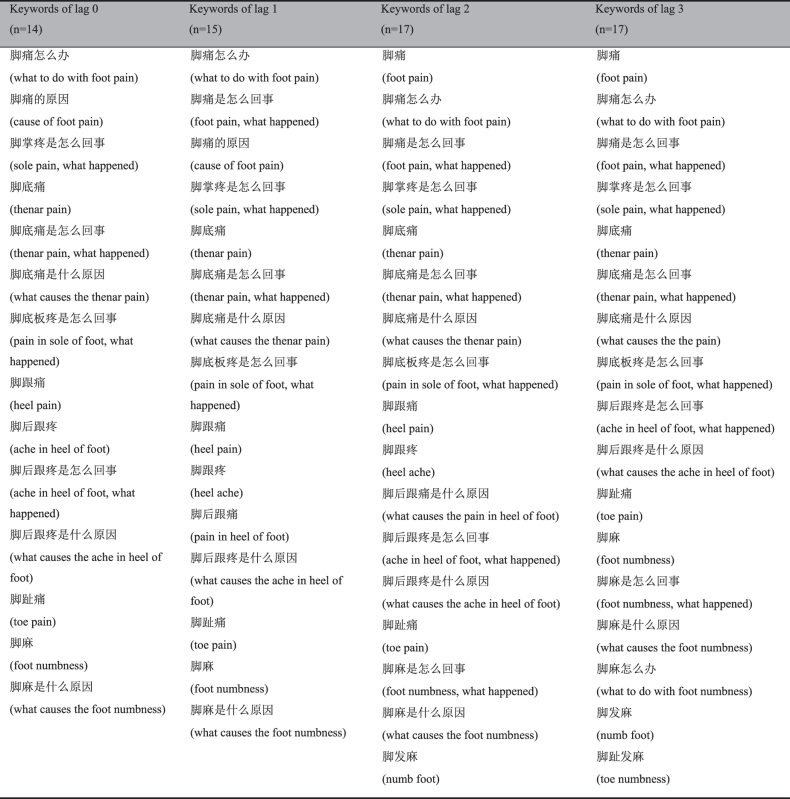

Baidu users search information in Chinese and the corresponding translation of each Chinese keywords are listed.

**Table 3:** Correlation between outbreak data and EM Search Index (lags of 0 to 3 days).

	**Lag 0**	**Lag 1**	**Lag 2**	**Lag 3**
Spearman’s rank correlation coefficient	0.747*	0.846*	0.863*	0.843*

^*^P < 0.0001.
