# Supplementary material for: Early detection of an epidemic erythromelalgia outbreak using Baidu search data
Source: Sci Rep. 2015 Jul 28;5:12649. doi: 10.1038/srep12649 (PMC4517510; doi:10.1038/srep12649)
Supplement: Supplementary Information [file srep12649-s1.pdf]

Early detecting epidemic erythromelalgia outbreak using Baidu search data

Yuzhou Gu[1], Fengling Chen[2], Tao Liu[1], Zhaoming Shao[1], Hualiang Lin[1], Xiaojuan Lv[1], Chaobin Liang[2], Weilin Zeng[1], Jianpeng Xiao[1], Yonghui Zhang[3], Cunrui Huang[4], Shannon Rutherford[5], Wenjun Ma[1*]

1. Guangdong Provincial Institute of Public Health, Guangdong Provincial Center for Disease Control and Prevention, Guangzhou, China

2. Chancheng Prefectural Center for Disease Control and Prevention of Guangdong Province, Foshan, China

3. Guangdong Provincial Center for Disease Control and Prevention, Guangzhou, China

4. School of Public Health, Sun Yat-sen University, Guangzhou, China

5. Center for Environment and Population Health, School of Environment, Griffith University, Brisbane, Australia

Correspondence to Dr. Wenjun Ma

Tel: +86-20-31051602

Fax: +86-20-31051352

Mailing address: No. 160, Qunxian Road, Panyu District, Guangzhou, China, 511430

E-mail address: mawj@gdiph.org.cn

Table S1 Keyword selection and first step filtering

Primary keywords<br>(n=19)	Website recommendation<br>(n=62)	First step filtering<br>(n=32)
脚痛<br>(foot pain)	脚痛<br>(foot pain)	脚痛<br>(foot pain)
	脚痛怎么办<br>(what to do with foot pain)	脚痛怎么办<br>(what to do with foot pain)
	脚痛是怎么回事<br>(foot pain, what happened)	脚痛是怎么回事<br>(foot pain, what happened)
	脚痛的原因<br>(cause of foot pain)	脚痛的原因<br>(cause of foot pain)
	脚痛是什么原因<br>(what causes the foot pain)	脚痛是什么原因<br>(what causes the foot pain)
	小孩脚痛<br>(child foot pain)	
	孕妇脚痛<br>(pregnant foot pain)	
	老人脚痛<br>(old people foot pain)	
	腰间盘突出引起脚痛<br>(slipped disc caused foot pain)	
	脚痛灸脚<br>(foot moxibustion for foot pain)	
	脚痛原因<br>(reason of foot pain)	
脚掌疼<br>(sole pain)	脚掌疼是怎么回事<br>(sole pain, what happened)	脚掌疼是怎么回事<br>(sole pain, what happened)
脚底痛<br>(thenar pain)	脚底痛<br>(thenar pain)	脚底痛<br>(thenar pain)
	脚底痛是怎么回事<br>(thenar pain, what happened)	脚底痛是怎么回事<br>(thenar pain, what happened)
	脚底痛是什么原因<br>(what causes the thenar pain)	脚底痛是什么原因<br>(what causes the thenar pain)
脚底很痛<br>(thenar very pain)	脚底很痛<br>(thenar very pain)	脚底很痛<br>(thenar very pain)
脚底板痛<br>(thenar ache)	脚底板痛<br>(thenar ache)	
	脚底板痛是什么病<br>(what disease is ache in pain)	
脚底板疼<br>(pain in sole of foot)	脚底板疼<br>(pain in sole of foot)	脚底板疼<br>(pain in sole of foot)
	脚底板疼是怎么回事<br>(pain in sole of foot what happened)	脚底板疼是怎么回事<br>(pain in sole of foot, what happened)
	走路脚底板<br>(pain in sole of foot when walking)	
	早上起床脚底板疼<br>(wake up with pain in sole of foot in the morning)	
脚底后跟痛<br>(pain in sole and heel)	脚底后跟痛<br>(pain in sole and heel)	
脚跟痛<br>(heel pain)	脚跟痛<br>(heel pain)	脚跟痛<br>(heel pain)
脚跟疼<br>(heel ache)	脚跟疼<br>(heel ache)	脚跟疼<br>(heel ache)
	脚跟疼是怎么回事<br>(heel ache, what happened)	脚跟疼是怎么回事<br>(heel ache, what happened)
足跟痛<br>(talalgia)	足跟痛<br>(talalgia)	足跟痛<br>(talalgia)
	足跟痛贴<br>(plaster for talalgia)	足跟痛是怎么回事<br>(talalgia, what happened)
	远洋足跟痛贴<br>(yuanyang plaster for talalgia)	
	足跟痛消贴<br>(pain - relieving plaster for talalgia)	
	足跟痛是怎么回事<br>(talalgia, what happened)	
足跟疼(heel sore)	足跟疼(heel sore)	

脚后跟痛<br>(pain in heel of foot)	脚后跟痛<br>(pain in heel of foot)<br>脚后跟痛是什么原因<br>(what causes the pain in heel of foot)<br>脚后跟痛是怎么回事<br>(pain in heel of foot, what happened)	脚后跟痛<br>(pain in heel of foot)<br>脚后跟痛是什么原因<br>(what causes the pain in heel of foot)
脚后跟疼<br>(ache in heel of foot)	脚后跟疼<br>(ache in heel of foot)<br>脚后跟疼是怎么回事<br>(ache in heel of foot, what happened)<br>脚后跟疼是什么原因<br>(what causes the ache in heel of foot)<br>脚后跟疼怎么办<br>(what to do with ache in heel of foot)<br>脚后跟疼怎么治疗<br>(how to treat the ache in heel of foot)	脚后跟疼<br>(ache in heel of foot)<br>脚后跟疼是怎么回事<br>(ache in heel of foot, what happened)<br>脚后跟疼是什么原因<br>(what causes the ache in heel of foot)
脚后跟疼痛<br>(sore in heel of fool)	脚后跟疼痛<br>(sore in heel of fool)	
后脚跟痛<br>(pain in back of the foot)	后脚跟痛是怎么回事(pain in back of the foot, what happened)	后脚跟痛是怎么回事(pain in back of the foot, what happened)
脚趾痛<br>(toe pain)	脚趾痛<br>(toe pain)<br>大脚趾痛<br>(big toe pain)	脚趾痛<br>(toe pain)
脚麻<br>(foot numbness)	脚麻<br>(foot numbness)<br>脚麻是怎么回事<br>(foot numbness, what happened)<br>脚麻是什么原因<br>(what causes the foot numbness)<br>脚麻了怎么办<br>(foot numbness, what to do)<br>脚麻怎么办<br>(what to do with foot numbness)<br>脚麻了<br>(foot numb)<br>脚麻的原因<br>(the reason of foot numbness)<br>手麻脚麻是怎么回事<br>(numbness in hand and foot, what happened)<br>青脚麻鸡<br>(Partridge Shank Chicken)<br>蹲久了脚麻<br>(foot numbness after long crouch)<br>手麻脚麻<br>(numbness in hand and foot)<br>糖尿病脚麻<br>(diabetes caused foot numbness)<br>经常脚麻<br>(recurring foot numbness)<br>小孩脚麻是怎么回事<br>(child foot numbness, what happened)	脚麻<br>(foot numbness)<br>脚麻是怎么回事<br>(foot numbness, what happened)<br>脚麻是什么原因<br>(what causes the foot numbness)<br>脚麻了怎么办<br>(foot numbness, what to do)<br>脚麻怎么办<br>(what to do with foot numbness)<br>脚麻的原因<br>(the reason of foot numbness)
脚发麻<br>(numb foot)	脚发麻<br>(numb foot)<br>经常脚发麻<br>(recurring numb foot)	脚发麻<br>(numb foot)
脚趾发麻<br>(toe numbness)	脚趾发麻<br>(toe numbness)<br>大脚趾发麻<br>(big toe numbness)	脚趾发麻<br>(toe numbness)

Baidu users search information in Chinese and the corresponding translation of each Chinese keywords are listed.

Table S2 Internet penetration of 31 provinces/municipalities of mainland China in 2013[14]

Province/Municipality	Internet population (10 million)	Internet penetration (%)
Beijing	1556	75.2
Shanghai	1683	70.7
Guangdong	6992	66.0
Fujian	2402	64.1
Tianjin	866	61.3
Zhejiang	3330	60.8
Liaoning	2453	55.9
Jiangsu	4095	51.7
Xinjiang	1094	49.0
Shanxi	1755	48.6
Qinghai	274	47.8
Hebei	3389	46.5
Hainan	411	46.4
Shaanxi	1689	45.0
Shandong	4329	44.7
Chongqing	1293	43.9
Neimenggu	1093	43.9
Ningxia	283	43.7
Hubei	2491	43.1
Jilin	1163	42.3
Heilongjiang	1514	39.5
Guangxi	1774	37.9
Xizang	115	37.4
Hunan	2410	36.3
Anhui	2150	35.9
Sichuan	2835	35.1
Henan	3283	34.9
Gansu	894	34.7
Guizhou	1146	32.9
Yunnan	1528	32.8
Jiangxi	1468	32.6
Total	61758	45.8
